# Selective Serotonin Reuptake Inhibitors and Adverse Effects: A Narrative Review

**DOI:** 10.3390/neurolint13030038

**Published:** 2021-08-05

**Authors:** Amber N. Edinoff, Haseeb A. Akuly, Tony A. Hanna, Carolina O. Ochoa, Shelby J. Patti, Yahya A. Ghaffar, Alan D. Kaye, Omar Viswanath, Ivan Urits, Andrea G. Boyer, Elyse M. Cornett, Adam M. Kaye

**Affiliations:** 1 Department of Psychiatry and Behavioral Medicine, Louisiana State University Health Science Center Shreveport, Shreveport, LA 71103, USA; haseeb.akuly@lsuhs.edu (H.A.A.); thanna@lsuhsc.edu (T.A.H.); 2 School of Medicine, Louisiana State University Health Shreveport, Shreveport, LA 71103, USA; cochoa@lsuhsc.edu (C.O.O.); spatt5@lsuhsc.edu (S.J.P.); yghaff@lsuhsc.edu (Y.A.G.); 3 Department of Anesthesiology, Louisiana State University Health Shreveport, Shreveport, LA 71103, USA; alan.kaye@lsuhs.edu (A.D.K.); ivanurits@gmail.com (I.U.); ecorne@lsuhsc.edu (E.M.C.); 4 College of Medicine-Phoenix, University of Arizona, Phoenix, AZ 85004, USA; viswanoy@gmail.com; 5 Department of Anesthesiology, Creighton University School of Medicine, Omaha, NE 68124, USA; 6 Valley Anesthesiology and Pain Consultants—Envision Physician Services, Phoenix, AZ 85004, USA; 7 Southcoast Physicians Group Pain Medicine, Southcoast Health, Wareham, MA 02571, USA; 8 Department of Psychiatry and Behavioral Sciences, Medical University of South Carolina, Charleston, SC 29464, USA; alg709@gmail.com; 9 Department of Pharmacy Practice, Thomas J. Long School of Pharmacy and Health Sciences, University of the Pacific, Stockton, CA 95211, USA; akaye@PACIFIC.EDU

**Keywords:** selective serotonin reuptake inhibitors, adverse effects, suicidality

## Abstract

Depression is the most prevalent psychiatric disorder in the world, affecting 4.4% of the global population. Despite an array of treatment modalities, depressive disorders remain difficult to manage due to many factors. Beginning with the introduction of fluoxetine to the United States in 1988, selective serotonin reuptake inhibitors (SSRIs) quickly became a mainstay of treatment for a variety of psychiatric disorders. The primary mechanism of action of SSRIs is to inhibit presynaptic reuptake of serotonin at the serotonin transporter, subsequently increasing serotonin at the postsynaptic membrane in the serotonergic synapse. The six major SSRIs that are marketed in the USA today, fluoxetine, citalopram, escitalopram, paroxetine, sertraline, and fluvoxamine, are a group of structurally unrelated molecules that share a similar mechanism of action. While their primary mechanism of action is similar, each SSRI has unique pharmacokinetics, pharmacodynamics, and side effect profile. One of the more controversial adverse effects of SSRIs is the black box warning for increased risk of suicidality in children and young adults aged 18–24. There is a lack of understanding of the complexities and interactions between SSRIs in the developing brain of a young person with depression. Adults, who do not have certain risk factors, which could be confounding factors, do not seem to carry this increased risk of suicidality. Ultimately, when prescribing SSRIs to any patient, a risk–benefit analysis must factor in the potential treatment effects, adverse effects, and dangers of the illness to be treated. The aim of this review is to educate clinicians on potential adverse effects of SSRIs.

## 1. Introduction

Depression is the most prevalent psychiatric disorder in the world, affecting 4.4% of the global population [1]. In the United States alone, the economic burden of the major depressive disorder increased by 21.5% from 2005 to 2015, when it was estimated to be USD 210.5 million/billion [2]. There are several types of depression and to differentiate there are specifiers that can be included. These are atypical features, anxious distressed, mixed features, melancholic features, psychotic features, catatonia, peripartum onset, and seasonal pattern [3]. Each different type of depression may respond better to a certain type of pharmacologic treatment than others. Despite an array of treatment modalities, depressive disorders remain difficult to manage due to many factors, including relatively high relapse rates while undergoing treatment and unfavorable side effect profiles of the medications available [4,5].

Preceding the discovery of selective serotonin reuptake inhibitors (SSRIs), monoamine oxidase inhibitors (MAOIs), and tricyclic antidepressants (TCAs) were the only options for pharmacologic intervention in depressive disorders. These drugs, however, had unfavorable side effect profiles, resulting in poor patient adherence. Consuming too much tyramine while on an MAOI may cause a potentially deadly hypertensive crisis. TCAs can cause blockage of cardiac sodium channels and cardiac arrhythmias [6].

Beginning with the introduction of fluoxetine to the United States in 1988, SSRIs quickly became a mainstay of treatment for a variety of psychiatric disorders. They were originally studied to target depression, but further investigation has led to their use in many anxiety disorders. SSRIs were not more effective than TCAs but had increased rates of patient adherence [7], largely due to their more favorable side effect profile. Despite being an immense step forward in the management of psychiatric disorders, SSRIs still have a variety of adverse effects that need to be reviewed and monitored.

The primary mechanism of action of SSRIs is to inhibit the presynaptic reuptake of serotonin at the serotonin transporter, subsequently increasing serotonin at the postsynaptic membrane in the serotonergic synapse [8]. Interestingly, the therapeutic effects of SSRIs cannot be entirely summed up by simple inhibition of serotonin transporter (SERT), and as such further mechanisms of action must be at work. A current theory posits that the neuronal stress caused by SSRIs causes a shift in brain homeostasis that results in downregulation of SERTs in some areas of the brain and upregulation in others [9]. This mechanism may explain why the full therapeutic effects of SSRIs are not realized until four to six weeks after initiation, despite significant immediate alterations in serotonin flux. The aim of this review to educate clinicians on potential adverse effects of SSRIs.

## 2. Current Uses of SSRIs

SSRIs are the first-line pharmacotherapy for most patients with depression because they are effective and generally better tolerated when compared to other antidepressants [8]. Though initially approved for the treatment of depression, the US Food and Drug Administration (FDA) has approved SSRIs for a variety of other conditions [8]. Clinically, prescribing SSRIs outside of their approved indications, or “off-label”, is increasing, as SSRIs demonstrate efficacy in several other therapeutic applications [10,11,12]. Off-label use of SSRIs can include for fibromyalgia, premature ejaculation, and neurocardiogenic syncope [13].

### 2.1. Depression

Depression is a debilitating illness that often interferes with a patient’s quality of life. It imposes a significant financial burden on a patient and the healthcare system, with both direct and indirect costs [14]. In 2018, a study reported increased use of outpatient services by patients with hypertension and/or diabetes with untreated depressive symptoms [15]. Along with addressing the patient’s depressive symptoms, treatment with antidepressants in these patients may decrease secondary health costs [15]. Additional comorbidities associated with depression are alcohol use disorder, anxiety disorders, and even somatoform disorders [16]. Adequate treatment of depression is thought to decrease these associated comorbidities as well.

The individual and societal impacts of depression highlight the importance of effective treatments. In patients who have not had a medication trial of an antidepressant, SSRIs are usually the first medication used in depression treatment. While SSRIs are the mainstay of pharmacological treatment for patients with depression, some patients do not respond to initial monotherapy and require the addition of other treatments [17]. One strategy involves combining SSRIs with psychotherapy. In one study, 30% of SSRI users utilized psychotherapy [18]. A meta-analysis that looked at studies that examined the use of antidepressants, psychotherapy, and both used in combination found that the use of the combination of antidepressants and psychotherapy offered better treatment outcomes which were still sustained two years later [19].

Additionally, SSRIs are often combined with other pharmacological agents to increase efficacy. In a Spanish survey, combination therapy was frequently prescribed by psychiatrists, with SSRI and mirtazapine being the most preferred combination [20]. Despite this, some research questions the clinical benefits and cost-effectiveness of SSRI combination therapy, and further studies are needed to fully elucidate what combination therapies are most beneficial for patients and when to use them [21,22].

### 2.2. Anxiety Disorders

Anxiety disorders, such as generalized anxiety disorder (GAD), social anxiety disorder (SAD), and panic disorder are some of the most common psychiatric disorders. SSRIs are currently the preferred medication for anxiety disorders due to an abundance of literature supporting their safety and effectiveness [23,24,25]. Compared to other anxiolytics, SSRIs have fewer side effects and treat depression, which is often comorbid with anxiety [26]. Results from a meta-analysis of 57 trials confirm that SSRIs are an effective treatment for anxiety disorders and that doses on the higher side of the therapeutic range are associated with greater symptom improvement [25].

### 2.3. Autism Spectrum Disorders

SSRIs are frequently and increasingly prescribed to individuals with autism spectrum disorders (ASD) [27]. Yet, a meta-analysis involving nine randomized control trials demonstrated insufficient evidence for the efficacy of the SSRIs fluoxetine, citalopram, and fluvoxamine in children with ASD [28]. In contrast, Reddihough et al. found that treatment with fluoxetine significantly decreased obsessive-compulsive behaviors in children and adolescents with ASD [29]. The uncertainty of SSRI efficacy in core autism behaviors highlights the need for further studies.

### 2.4. Eating Disorders

SSRIs have demonstrated to be useful treatment options for bulimia nervosa (BN) and binge eating disorder (BED) [30]. Fluoxetine is the only SSRI with FDA approval for BN, though other SSRIs have shown effectiveness [30,31,32]. In BED, a meta-analysis of 45 studies showed SSRIs provided some improvement in remission and reduction in the frequency of binge eating but no improvement in patient BMI [33].

### 2.5. Premenstrual Syndrome/ Premenstrual Dysmorphic Disorder

SSRIs are an essential pharmacological treatment for patients with unmanageable premenstrual syndrome (PMS) and premenstrual dysmorphic disorder (PMDD) [34]. SSRIs can be taken either continuously or in the luteal phase to reduce the symptoms of PMS and PMDD [35]. Most SSRIs exhibit equal efficacy for the treatment of PMS and PMDD, so a provider’s choice of SSRI should be based on anticipated side effects and the patient’s response to the drug [34,35,36].

### 2.6. Menopausal Vasomotor Symptoms

Traditionally, hormone replacement therapy has been used for vasomotor symptoms associated with menopause; however, safety concerns and poor compliance have led to the use of alternative medications. SSRIs, especially paroxetine, can be effective for managing menopausal vasomotor symptoms, reducing both the frequency and severity of hot flashes in menopausal women [37,38,39].

### 2.7. Myocardial Infarctions

Some, but not all, evidence links SSRIs to a reduced risk of myocardial infarctions (MI), especially in patients with depression and a cardiovascular history [40,41,42,43,44]. For example, in a study of Veteran’s Affairs (VA) patients with depression, 12 weeks of SSRI therapy significantly decreased the risk of MI and mortality [45]. Although the exact mechanism is unknown, some studies hypothesize that SSRIs may reduce the likelihood of MI by inhibiting serotonin-mediated platelet activation [46,47,48]. It should also be noted that not all SSRIs are safe to use in patients with cardiac disorders and should not be used as a treatment for cardiac concerns alone.

### 2.8. Nociceptive Pain

Pharmacological relief for nociceptive pain typically involves opioids. However, the clinical utility of these drugs can be challenging due to the development of tolerance and dependence [49,50]. The use of the SSRI fluoxetine has been evaluated as an adjunctive therapy option for the management of nociceptive pain. Animal studies indicate that co-administration of fluoxetine with morphine prevents the development of tolerance and dependence [51,52]. Alboghobeish et al. found that fluoxetine increased the antinociceptive effect of morphine, in addition to attenuating tolerance and withdrawal [52].

### 2.9. Gastrointestinal Disorders

Gastrointestinal (GI) disorders are frequently comorbid in patients with depression and anxiety disorders [53,54,55]. Serotonin transporters are located on neurons, glial cells, blood platelets, and enterocytes, and altered signaling of serotonin in the gut may contribute to the symptoms associated with GI disorders [56,57]. Antidepressants have shown to have anti-ulcerative effects and have been increasingly prescribed in those with GI disorders [58]. Multiple animal studies have shown that the SSRI fluvoxamine protects against the development of peptic ulcers through antioxidant and anti-inflammatory mechanisms [59,60]. Certain SSRIs may improve symptoms of irritable bowel syndrome (IBS); however, their efficacy is controversial, so they should only be prescribed in IBS patients with comorbid depression or anxiety [61,62].

## 3. SSRIs: Types and Differences

The six major SSRIs that are marketed in the USA today, fluoxetine, citalopram, escitalopram, paroxetine, sertraline, and fluvoxamine, are a group of structurally unrelated molecules that share a similar mechanism of action. Even though their primary mechanism of action is similar, each SSRI has unique pharmacokinetics, pharmacodynamics, side effect profile, and efficacy that dispose each to be more or less suited for a particular clinical niche. Selecting the right SSRI depends upon the individual patient and whether or not the side effects can be utilized as secondary therapeutic effects.

With regards to adverse effects, many are shared among all SSRIs to varying degrees, including sexual dysfunction, gastrointestinal distress, prolonged QT interval, and xerostomia [63]. Some of these side effects will be discussed further in later sections, but they are also important delineating factors among the different SSRIs.

### 3.1. Fluoxetine

Fluoxetine, sold most commonly under the brand names Prozac and Sarafem, is the oldest and best-studied of the SSRIs. Sarafem is only FDA-approved for use in premenstrual dysphoric disorder, but other forms of fluoxetine are currently approved for use in major depressive disorder, bipolar disorder, BN, obsessive-compulsive disorder (OCD), panic disorder (PD), and treatment-resistant depression.

Among the SSRIs, fluoxetine exhibits the least specific binding to SERT and, at high doses, can increase synaptic norepinephrine and dopamine levels [64]. Fluoxetine tends to be associated with higher rates of weight loss, agitation, and anxiety when compared to other SSRIs, which may be related to its slightly reduced binding specificity [65,66]. Despite being the least specific SSRI for SERT, fluoxetine exhibits much higher binding specificity than TCAs and MAOIs, resulting in a much more favorable side effect profile.

### 3.2. Citalopram/Escitalopram

Citalopram and escitalopram are both FDA-approved in the United States for use in treating major depressive disorder (MDD), while escitalopram is also approved for GAD. The drug citalopram is the racemic mixture of both R and S enantiomers of citalopram, while escitalopram is only the S enantiomer. The S enantiomer is the compound of interest when treating depression, while the R enantiomer appears to have no effect and may even interfere with the effects of its racemate [67]. Due to its lack of the R enantiomer, escitalopram may be more efficacious than citalopram for depression and has the highest specificity for SERT of the SSRIs [68,69].

Both drugs, but especially citalopram, are more often associated with QT prolongation than other SSRIs, and this relationship appears to be dose-dependent [70]. As of March 2021, the FDA issued a safety communication regarding these two medications about the QT elongation. It cited a study that found more profound changes in the QTc that started at 40 mg of citalopram [71]. At 20 mg, the QTc was changed by 8.5 ms. At 40 mg the QTc was changed by 12.6 ms, and at 60 mg the QTc was changed by 18.5 ms [71]. It is because of this study that the FDA recommends to not given doses above 40 mg of citalopram.

### 3.3. Paroxetine

Paroxetine is currently approved for MDD, GAD, post-traumatic stress disorder, and PMDD, among others. Of the SSRIs, it inhibits SERT most potently [72]. One study found that paroxetine and fluoxetine both inhibited the enzyme cytochrome P450 2D6 (CYP2D6), required for the conversion of tamoxifen to its more active metabolites, resulting in lower levels of these metabolites and, potentially, a reduction in the drug’s anticancer effects [73]. This interaction warrants further investigation.

### 3.4. Fluvoxamine

Sold most commonly under the brand name Luvox, fluvoxamine is approved in the United States for the treatment of obsessive-compulsive disorder and SAD. Like other SSRIs, fluvoxamine demonstrates high specificity for SERT; however, unlike other SSRIs, it is also a potent agonist at the sigma-1 receptor [74]. The role of the sigma-1 receptor is not completely understood, but it is thought to be involved in some of the cognitive improvements seen with fluvoxamine therapy [75]. Fluvoxamine is also the only monocyclic SSRI, with the others featuring two to four rings in their chemical structures [76].

### 3.5. Sertraline

Sertraline is approved for MDD, OCD, PD, PTSD, SAD, and PMDD. In one review, sertraline was found to be more efficacious in the acute phase (between six to twelve weeks) of treatment of MDD than other SSRIs [77]. The same review also found sertraline to be associated with a higher rate of diarrhea as a side effect. Sertraline may demonstrate antagonist activity at the sigma-1 receptor but with a lesser affinity than fluvoxamine as an agonist [74].

## 4. Pharmacokinetics/Pharmacodynamics

As discussed previously, inhibition of presynaptic SERT causes an increase in serotonin at the synaptic cleft leading, in part, to the therapeutic effects of SSRIs and other antidepressants. SSRIs marked a massive improvement in the treatment of depression for many reasons, particularly their binding specificity. Compared to MAOIs and TCAs, SSRIs have much higher specificity for SERT, which allows them to avoid many of the antimuscarinic, antihistaminergic, and antiadrenergic side effects of TCAs [78]. Among the SSRIs, escitalopram has the highest specificity of all and is almost twice as effective at inhibiting SERT as its counterpart citalopram [79].

Half-lives of the SSRIs under steady state conditions depends on the individual drug. For example, the half-life of fluoxetine is between one and four days [80]. Paroxetine has a half-life around 21 h and citalopram has a half-life around 26 h. Sertraline and citalopram show linear pharmacokinetics while fluoxetine, fluvoxamine, and paroxetine show nonlinear pharmacokinetics [80].

The SSRIs are metabolized by the cytochrome P450 system in the liver. Fluvoxamine, fluoxetine, and sertraline inhibit certain cytochrome P450 enzymes to a great degree, which may cause more drug–drug interactions than found with citalopram and escitalopram [81,82,83,84]. One study showed that fluoxetine, paroxetine, and possibly fluvoxamine inhibit their own metabolism, which may result in nonlinear kinetics at higher doses [85]. This does not increase their rate of adverse effects, but care should be taken when prescribing these drugs to a patient with impaired drug elimination due to liver disease, kidney disease, or advanced age [86]. Fluoxetine has the longest half-life of the SSRIs—between four and six days for the compound itself, and seven to fifteen days for its active metabolite norfluoxetine [65,87]. Sertraline and citalopram exhibit linear kinetics and so may be better choices when initiating therapy for an individual with impaired drug elimination [65]. All of the SSRIs inhibit CYP2D6 with fluoxetine and paroxetine being the most potent inhibitor [88]. Interactions with CYP3A4 appear to not be significant with SSRIs [88].

## 5. SSRIs and Adverse Effects

SSRIs are generally better tolerated than other antidepressants, but common side effects may include nausea, vomiting, insomnia, drowsiness, headache, decreased sex drive, and agitation [8,89]. Here, we will discuss some of the less common adverse effects of SSRIs reported in literature, with a focus on extrapyramidal symptoms (EPS), serotonin syndrome, QT prolongation, rash, birth defects, hyponatremia, and cataracts.

### 5.1. Extrapyramidal Symptoms

Although uncommon, EPS in patients treated with SSRIs has been observed in numerous studies [90,91,92]. One study identified 86 case reports connecting the use of SSRIs with the development of dystonia, parkinsonism, dyskinesia, and akathisia. Most of these cases occurred within 30 days of treatment initiation or dose increase, with citalopram, escitalopram, fluoxetine, and sertraline most frequently involved [90]. This association highlights the importance of monitoring patients during SSRI therapy for the development of EPS [90,91,92].

### 5.2. Serotonin Syndrome

Serotonin syndrome is a potentially fatal consequence of serotonergic overactivity in the peripheral and central nervous systems [93]. Though rare, incidence is increasing due to widespread SSRI use [93]. The majority of cases involve a combination of serotonergic drugs, though SSRI monotherapy may also lead to serotonin syndrome [94,95]. One case reported moderate serotonin syndrome involving hyperreflexia and ankle clonus in an adult male on sertraline monotherapy [96]. Most other cases of serotonin syndrome with SSRI monotherapy have involved overdose or switching SSRI therapy without cross-titration [97,98,99]. To prevent serotonin syndrome in patients on SSRIs, providers should exercise caution when combining, switching, or discontinuing these drugs [100].

### 5.3. QT Prolongation

SSRIs, especially citalopram, can prolong the QT interval by antagonizing myocyte potassium channels, which may trigger fatal reentrant tachycardias, such as Torsades de Pointes [101,102]. Rochester et al. concluded that citalopram considerably increases the risk of QT prolongation in patients over 60 years old [102]. The risk of QT prolongation with citalopram seems to be dose-dependent, and those with cardiac disease and other QT-prolonging risk factors are more vulnerable [103]. Conversely, data from a retrospective review found no association between QT prolongation and citalopram (or escitalopram) in geriatric patients. The authors suggest clinicians compare the risk of QT prolongation versus the clinic risk of lowering citalopram/escitalopram dose on a case-by-case basis until further prospective studies are completed [104]. The FDA recommends a maximum 20 mg daily dose of citalopram and ECG monitoring for at-risk and older individuals [71,102].

### 5.4. Rash

SSRIs have been associated with photosensitivity, spontaneous bruising, pruritus, alopecia, and urticaria [105]. In 2019, a female with a history of asthma and an autoimmune disorder developed an itchy pruritic rash four weeks after starting escitalopram [106]. In another case report, fluoxetine was thought to be the cause of acute urticaria and angioedema in a 23-year-old male [107]. Rarely, serious cutaneous reactions such as Stevens–Johnson syndrome, toxic epidermal necrosis, erythema multiforme, acute generalized exanthematous pustulosis, and drug-induced hypersensitivity reactions have been reported in patients taking SSRIs [108,109,110]. These conditions may be life-threatening, stressing the importance of patient and provider education in recognizing adverse cutaneous reactions early [108].

### 5.5. Congenital Malformations

Given the widespread use of SSRIs and their ability to cross the placental barrier, numerous studies have evaluated the association between SSRIs and congenital defects, with inconsistent findings [111]. More recent data report an association between SSRIs (mainly paroxetine and fluoxetine) and a slightly increased risk of congenital defects, particularly cardiac malformations [111,112,113,114]. Literature suggests that SSRIs may potentially interfere with serotonin signaling important for myocardial development in the developing fetus [112,113,115]. However, the discontinuation of SSRI treatment may present an even greater risk for fetal development and the health of the mother, and thus, decisions on pharmacotherapy in pregnant women should be individualized [116,117].

### 5.6. Hyponatremia

Among all antidepressants, SSRIs carry the highest risk of hyponatremia, especially in the initial weeks of treatment [118,119]. Although the mechanism is unknown, serotonin may increase antidiuretic hormone, thereby inducing syndrome of inappropriate secretion of antidiuretic hormone (SIADH) [119,120]. Most cases of hyponatremia due to SSRIs involve elderly patients, but other risk factors include concomitant use of hyponatremia-inducing drugs, low body weight, female gender, low serum sodium, and severe illness [120,121,122].

### 5.7. Cataracts

Recent studies suggest a positive association between long-term use of SSRIs and the development of cataracts. A nested case-control study and a meta-analysis found that continuous use of fluoxetine and fluvoxamine significantly increased the risk of cataracts [123]. In contrast, Becker et al. concluded that SSRI therapy does not alter the risk of cataracts [124]. Given these inconsistent findings, providers should recommend regular eye examinations for patients on SSRIs [125].

## 6. Suicidal Ideation

According to the Centers for Disease Control and Prevention, suicide is the tenth leading cause of death [126]. Suicidal ideation (SI) is defined as thinking about suicide or taking one’s own life, and Table 1 shows the warning signs of SI. Psychiatric illnesses, especially major depressive disorder (MDD), are closely linked to suicide. Beginning in the 1950s, TCAs, including amitriptyline, amoxapine, desipramine (Norpramin), doxepin, imipramine (Tofranil), nortriptyline (Pamelor), protriptyline, and trimipramine, were the first line of treatment for MDD. TCAs were used widely until SSRIs were introduced on the market in 1982 and became first-line due to their direct action and more favorable side effect profile. One of the most worrisome side effects of treatment with antidepressants, especially SSRIs, is new or worsening SI.

Treatment Emergent Suicidal Ideation (TESI) is the increased likelihood of developing SI after beginning treatment with an SSRI or other antidepressant [127]. Clinicians have variably reported suspicion that antidepressants may increase or decrease suicidal ideation and/or behaviors in patients. Clinical studies have a mix of predictive outcomes, including direct, indirect, or no effect of SSRIs on SI. Although studies have been conducted worldwide, subjectivity in defining SI has contributed to their inconclusivity in adults.

### 6.1. Adolescents

In 2004, the US Food and Drug Administration (FDA) added a black box warning level 5 to all antidepressants of suicidality for children and young adults aged 18–24 years [128]. Anxiety, agitation, hostility, restlessness, or impulsive behavior in adolescents after starting an antidepressant may be the natural course of worsening depression or TESI [129]. Following this, physicians began to underdiagnose MDD in adolescents and prescribed them fewer antidepressants, and patients under age 18 were often not included in studies.

In 2014, a retrospective cohort study investigated 36,842 children aged 6 to 18 years old, with a mean age of 14 [130]. The children were enrolled in Tennessee Medicaid between 1995 and 2006 and were all new users of one antidepressant medication, including fluoxetine, sertraline, paroxetine, citalopram, escitalopram, or venlafaxine [130]. Four hundred nineteen cohort members who had a medically treated suicide attempt with explicit or inferred attempt to die, confirmed through medical record review, including four who completed suicide [130]. Compared to the national suicide average in adolescents, there was no evidence of increased risk for serious suicide attempts on any of the individual antidepressants [130]. One limitation of this study was the focus on suicide attempts, thereby possibly missing some SI.

A European multicenter double-blind study investigated 244 adolescents aged 13 to 18 years old with MDD who were randomized into a placebo group (*n* = 124) or treatment with citalopram (*n* = 120) [131]. More than two-thirds of the adolescents received psychotherapy, and one-third of both groups withdrew. The adolescents were measured with the Kiddie Schedule for Affective Disorders and Schizophrenia (K-SADS) Present score, and the Montgomery Asberg Depression Rating Scale (MADRS). For patients not receiving psychotherapy, there was a higher percentage of K-SADS-P responders with citalopram (41%) versus placebo (25%), and a significantly higher percentage of MADRS responders and remitters with citalopram (52% and 45%, respectively) versus placebo (22% and 19%, respectively) [131]. Five patients in the placebo group and fourteen patients in the citalopram group experienced suicide attempts, defined to include SI and suicidal tendencies, which was not a significant difference [131]. However, the K-SADS-P SI single item showed a worsening in the placebo (18%) than in the citalopram group (8%) [131]. This study highlights the difficulty determining a clear correlation between SI and SSRIs, even in adolescents, and the need for further studies.

### 6.2. Adults

Given the completed development of the brain, and therefore, the greater social acceptability of studies in adults, the relationship between SI and SSRIs in adults is better characterized.

From July 2001 to April 2004, Sequenced Treatment Alternatives to Relieve Depression (STAR*D) enrolled 4041 18–75-year-old outpatients diagnosed with nonpsychotic MDD. The group was representative of the demographics of the US Census [132]. The patients were evaluated using the Quick Inventory of Depressive Symptoms (QIDS-SR) and received treatment with citalopram for 12 weeks. The dosing schedule started at 20 mg/day, increased to 40 mg/day at week four, and increased to 60 mg/day at week six. Of the 1909 participants with SI at baseline, 1738 returned for treatment visits, and 74% of those improved from baseline, while only 4% had worsened SI [132]. In the small minority who experienced worsening SI during the study, most improved by their last treatment visit [132]. This study demonstrated a clear improvement of SI in depressed adults treated with citalopram, though there was no comparison placebo group.

In 2009, there was a government-run study of 1014 adults with MDD in a real-world inpatient setting [133]. The Hamilton Depression Rating Score (HAMD) was used to evaluate the patients who were treated with an SSRI, a TCA, or placebo. Those treated with fluoxetine, showed a 72% improvement in SI, those treated with the TCA showed a 69.8% improvement, and the placebo group showed a 54% improvement [133]. This study showed a clear improvement in SI with an SSRI or a TCA [133]. One limitation was a potential selection bias, as the participants were inpatients and not randomly selected. Depressed patients were rarely discharged when suicidal, and the final evaluation was completed at discharge [133].

In 2018, the Arzneimittelsicherheit in der Psychiatrie (AMSP) program for Drug Safety in Psychiatry studied 219,635 patients from 1993 to 2014 to determine the correlation between SI and antidepressants, including SSRIs, TCAs, serotonin-norepinephrine reuptake inhibitors (SNRIs), and noradrenergic and specific serotonergic antidepressants (NaSSAs). There were 83 documented suicidal cases during the study—44 cases of SI, 34 attempted suicides, and 5 fatal suicides—with an incidence rate of 0.04% [134]. Increased restlessness, ego-dystonic thoughts or urges, and impulsivity contributed to suicidality [134]. This study found a rare and not clinically significant association between antidepressant use and SI [134].

### 6.3. Potential Confounders

In adults, specific symptoms, such as insomnia, activation, and anxiety during antidepressant treatment, may increase SI [135]. Being Hispanic or African American, taking sedative medications, abusing alcohol or drugs, increased depression severity, melancholic features, absence of hypersomnia, and comorbidity are associated with increased SI during treatment [128]. Woman are slightly less likely than men to develop SI and tend to develop it later in treatment [128]. Being widowed, better work performance, weight loss, and the presence of vascular or neurologic comorbidities were associated with worsening SI [128]. Clinical trials that control for these variables, are needed to further determine the direct effect of SSRIs on SI.

## 7. Conclusions

This review discussed SSRIs and their clinical indications, adverse effect profiles, within-class differences, and potential association with SI. Despite being initially developed for the treatment of depressive disorders, SSRIs are now used in anxiety disorders, other psychiatric disorders, and certain medical conditions. Each SSRI possesses unique characteristics that make it a better fit for certain patients, depending on patient comorbidities and genetics, and potential adverse effects. For patients who require multiple pharmacologic interventions for various ailments, an SSRI that does not inhibit cytochrome P450 enzymes, such as citalopram or escitalopram, may be considered in order to avoid drug–drug interactions.

While SSRIs are much better tolerated than their predecessors, the TCAs and MAOIs, there are potential adverse effects. Some of the more common ones include GI upset, insomnia, agitation, and sexual dysfunction. One of the more controversial adverse effects of SSRIs is the black box warning for increased risk of suicidality in children and young adults aged 18–24. There is a lack of understanding of the complexities and interactions between SSRIs in the developing brain of a young person with depression. Adults, who do not have certain risk factors, as stated previously, do not seem to carry this increased risk of suicidality. Ultimately, when prescribing SSRIs to any patient, a risk–benefit analysis must factor in the potential treatment effects, adverse effects, and dangers of the illness to be treated.

## Figures and Tables

**Table 1:** Symptoms of Suicidal Ideation.

Behavioral Symptoms	Psychosocial Symptoms	Physical Symptoms	Cognitive Symptoms
Giving away possessions	Helplessness	Scars	Preoccupation with death
Talking about death	Psychosis	Altered sleeping habits	Believing suicide is only way to end emotional pain
Getting affairs in order	Self-loathing	Altered eating habits	
Saying goodbye to loved ones	Hopelessness	Terminal illness	
Obtaining suicidal items	Paranoia		
Decreasing social contact	Emotional Pain		
Increasing drug/alcohol use	Mood swings		
Withdrawing	Personality changes		
Increasing risky behavior	Severe anxiety		

## Data Availability

No data were collected in this manuscript but all were cited as appropriate and can be found in the reference section.
